# Extraordinarily high serum CA 19-9 in setting of pancreatic necrosis and underlying pancreatic adenocarcinoma: a case report

**DOI:** 10.1093/jscr/rjad550

**Published:** 2023-10-14

**Authors:** Ashlyn McConnell, Tyler Stoneman, Stanley Hewlett

**Affiliations:** Department of Surgery, Princeton Baptist Medical Center, Birmingham, AL 35211, United States; Edward Via College of Osteopathic Medicine, Spartanburg, SC 29303, United States; Department of Surgery, Princeton Baptist Medical Center, Birmingham, AL 35211, United States

**Keywords:** pancreatic cancer, CA 19-9, jaundice, tumor markers

## Abstract

Carbohydrate antigen (CA 19-9) is the most validated marker for both sensitivity and specificity of pancreatic adenocarcinoma used to aid diagnosis of symptomatic patients as well as to evaluate the progression or treatment of disease. Though higher levels of CA 19-9 tend to correlate with neoplastic disease, elevated levels are also often seen in patients with benign gastrointestinal diseases, such as obstructive jaundice and pancreatitis. We present a case of a 74-year-old male who was admitted for abdominal pain and worsening jaundice who was diagnosed with extensive pancreatic necrosis and an underlying invasive pancreatic adenocarcinoma whose serum level of CA 19-9 was found to be extraordinarily high.

## Introduction

Carbohydrate antigen (CA 19-9) is the most validated marker for both sensitivity and specificity of pancreatic adenocarcinoma used in diagnosis of symptomatic patients as well as to evaluate the progression or treatment of disease [1]. It is produced by ductal cells in the pancreas and biliary system, as well as by epithelium of the stomach, uterus, colon, and salivary glands. The normal range for CA 19-9 is from 0 to 37 U/ml. Levels can be overexpressed in benign diseases, though they tend to present lower compared with their malignant counterparts, and are often normalized following resolution of the condition [2, 3]. In contrast, malignant pathologies are associated with remarkably increased serum CA 19-9 levels [1, 2, 4]. Notably, studies have established a positive correlation between CA 19-9 and tumor size, burden and pathological stage [5]. Despite its common clinical use in pancreatic neoplastic cases, this protein is simply associated with, rather than specific to, tumors or malignancies, and its utility should serve as an adjunct to radiological and other clinical information in the process of forming a diagnosis.

The aim of this article was to present a case of an extraordinarily high serum CA 19-9 level found in a patient diagnosed with pancreatic necrosis and underlying pancreatic tail adenocarcinoma, and to explore possible mechanisms for such drastic serum elevations.

## Case report

A 74-year-old previously healthy male patient presented to the emergency department with complaints of worsening abdominal pain and jaundice. He endorsed loss of appetite, intermittent nausea, weakness, increasing pruritus, and an unintentional weight loss of 40 pounds over the past 5 months. His medical history was significant only for hypertension and type 2 diabetes mellitus on Ozempic. He denied any history of pancreatitis, gastritis, tobacco, alcohol, or drug use. He cited no known personal or family history of cancer.

Laboratory data on admission demonstrated absent leukocytosis, transaminitis (alkaline phosphatase: 525 U/L [normal range: 44–147 U/L], aspartate aminotransferase 141 U/L [normal range: 8–33 U/L], alanine transaminase 205 U/L [normal range: 7–55 U/L]), hyperbilirubinemia (total bilirubin 4 mg/dl [normal range: 0.1–1.2 mg/dl], direct bilirubin 3.3 mg/dl [normal range: <0.3 mg/dl]), and a normal lipase (140 U/L [normal range: 0–160 U/L]). Tumor markers were notable for carcinoembryonic antigen 289 ng/ml and CA 19-9 579 883 U/ml.

**Figure 1:**
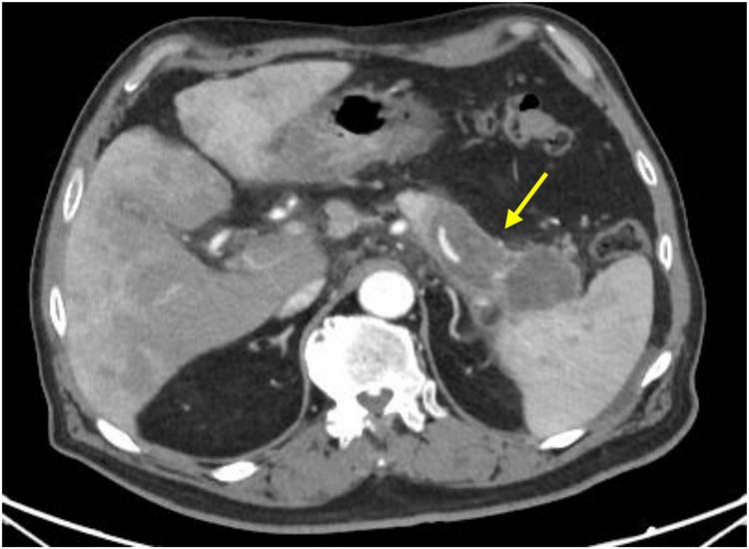
CT scan with IV contrast: axial image showing hypoenhancing infiltrative pancreatic tail mass with central necrosis (arrow), measuring 8 × 3 × 4 cm with adjacent stranding and mass abutting the spleen that appears to completely encase the splenic artery and vein.

**Figure 2:**
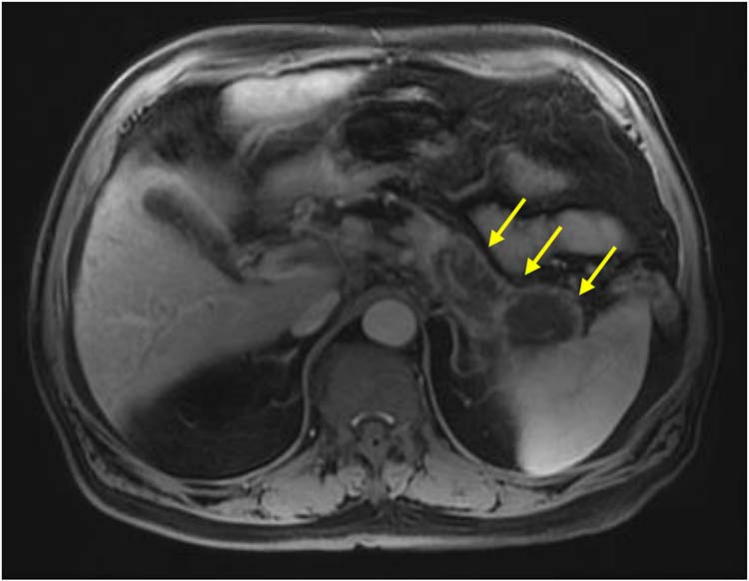
MRI abdomen (MRCP) with IV contrast: axial image showing diffuse enlarged hypointense signal of pancreatic body and tail on T1 weighted imaging encircled by a thin rim of enhancement representing normal pancreatic tissue (arrows).

Initial imaging with computed tomography (CT) of the chest, abdomen, and pelvis with IV contrast identified a hypoenhancing infiltrative pancreatic tail mass with central necrosis, as seen in Fig. 1. Given the diffuse hypodensity and without evidence of metastatic disease, findings were favored to represent pancreatitis with pancreatic necrosis. Magnetic resonance cholangiopancreatography (MRCP) was subsequently obtained which again revealed findings favored to be pancreatic necrosis rather than malignancy, as seen in Fig. 2. He then underwent an endoscopic retrograde cholangiopancreatography, which found a common bile duct stone which was extracted, as well as a high-grade benign-appearing ampullary stenosis, which was brushed and stented. The patient’s hyperbilirubinemia unfortunately failed to resolve following biliary ductal decompression. His highest documented total bilirubin was 24 mg/dl, largely from a direct component. Finally, he underwent an endoscopic ultrasound (EUS), which identified a mass in the body and tail of the pancreas advancing into the second portion of the duodenum with extensive surrounding ascites. Two fine-needle biopsies were taken, which resulted positive for invasive adenocarcinoma.

The patient experienced a stark decline over the next several days following EUS, demonstrated by worsening renal and liver function, confusion, and weakness. Ultimately the patient was discharged home to hospice and passed 5 days later, ~4 weeks after initial hospital presentation.

## Discussion

The patient discussed above presented with a history concerning for malignant disease in the setting of vague abdominal pain, weakness, weight loss, and worsening jaundice. His pancreatic lesion was characterized as probable necrosis on all imaging reports; however, he clinically had limited history to support the diagnosis. His elevated CA 19-9 was initially attributed to a benign obstructive hepatobiliary etiology, but in retrospect, was likely an additive effect stemming from both benign and malignant processes, including diabetes, choledocholithiasis, and highly advanced pancreatic tail adenocarcinoma.

In a cross-sectional study by Gul *et al.* [6], patients with diabetes mellitus often have higher baseline levels of CA 19-9 than those without diabetes mellitus. Though the mechanism is unclear, some autopsy studies showed changes to pancreatic exocrine glands including atrophy, tissue inflammation, and reduced weight [7]. Choledocholithiasis is another benign condition where elevated CA 19-9 can be seen. In an article written by Ghallab *et al*. [4], CA 19-9 levels ranged from 250 U/ml to over 98 000 U/ml in patients with benign obstructive pathology, possibly secondary to leakage of CA 19-9 into systemic circulation. Importantly though, studies have illustrated rapid normalization of CA 19-9 after intervention [2, 3].

Interestingly, Ritts *et al*. [8] reported that of the patients with a malignant pancreatic tumor, 78% expressed elevated CA 19-9 antigen levels, and 56% of those had levels greater than 500 U/ml. Furthermore, it has been studied that in patients with pancreatic adenocarcinoma whose CA 19-9 levels reached above 1000 U/ml, 96% were found to be unresectable [1].

In conclusion, this case presents a rare manifestation of invasive pancreatic body and tail adenocarcinoma hidden inside pancreatic necrosis causing an extraordinarily elevated production of CA 19-9. It highlights the need for imaging and laboratory results to be individualized according to the clinical scenario of each patient, and to promote further investigation when abnormal test results occur.
